# The Effect of Screen Exposure and Early Language Stimulation on Language and Social Development of Children

**DOI:** 10.7759/cureus.113133

**Published:** 2026-07-21

**Authors:** Vimalraj Vijayakumar, Arunkumar T, Karthick Prasaad R, Suresh Rangaraj, Sridharini K, Hariharan G

**Affiliations:** 1 Department of Pediatrics, SRM Medical College Hospital and Research Centre, SRM Institute of Science and Technology, Chengalpattu, IND

**Keywords:** early language stimulation, language development, pediatric screen time, screen exposure, social development

## Abstract

Introduction

Excessive screen exposure during infancy and early childhood is linked to delayed language and social milestones. Prospective observational data regarding how natural, self-selected variations in screen reduction and caregiver-led language stimulation impact real-world milestones development remain limited.

Aims and objectives

The aim of this study is to prospectively evaluate the association between changes in screen exposure and caregiver language stimulation on language and social development in nine- to 18-month-old children who were initially exposed to excessive screen time.

Methodology

In this prospective cohort study, 72 children aged 9-18 months with a baseline screen exposure of more than two hours/day were followed up in a tertiary care hospital. During the six-month follow-up, we did not implement interventions or prescribe treatments but rather observed families and stratified them into two exposure cohorts based on validated monthly lifestyle diaries: Cohort A (spontaneous screen reduction to less than one hour/day plus natural early language stimulation by “serve-and-return” interactions, n=36): and Cohort B (spontaneous screen reduction only without structured language stimulation routines, n=36). Development was assessed using the Language Evaluation Scale Trivandrum (LEST) and Home Screen Questionnaire (HSQ) at baseline and at six months. We used multivariable linear regression to adjust for baseline confounders, including parental education and household socioeconomic status.

Results

At six-month follow-up, Cohort A infants had a significantly greater mean increase in overall LEST+HSQ scores than Cohort B (+16.3 vs. +7.4, p<0.001). After controlling for baseline demographics, Cohort A had significantly higher language scores in the independent domain (30.2±3.4 vs. 24.8±3.9, p<0.001) and social communication scores (28.2±3.1 vs. 24.4±3.5, p<0.001) than Cohort B.

Conclusions

Combining natural caregiver interaction with structured language stimulation and reduced screen time is much more associated with better language and social development outcomes than reducing screen time alone. Clinicians should emphasize the promotion of increased caregiver interaction during early childhood along with screen time restriction rather than screen restriction alone.

## Introduction

The early years of childhood are critical for neurodevelopment, characterized by rapid synaptogenesis, axonal myelination, and extreme environmental plasticity [1]. The language and socio-emotional milestones achieved in this phase depend largely on rich, two-way communication between caregiver and child, sometimes referred to as “serve-and-return” interactions [2]. This is the critical period in which environmental stimulation determines cognitive and linguistic outcomes [2].

Digital devices are ubiquitous, and media exposure is at an all-time high at an early age [2]. Epidemiological data show a dose-dependent inverse relation between extended screen time and toddler expressive vocabulary [2]. Passive media consumption often supplants crucial human interactions like shared reading, storytelling, singing, and conversational give-and-take, all of which are necessary to reinforce language processing networks in the developing brain [2].

Current recommendations from the American Academy of Paediatrics (AAP) and the World Health Organization (WHO) recommend total avoidance of passive screen media in children younger than 18-24 months of age [2]. There have been clinical trials of prescribed behavioural interventions, but no prospective cohort data on the effects of self-selected naturally occurring parental lifestyle adaptations such as screen reduction and spontaneous home language enrichment on real-world developmental trajectories [2]. This prospective cohort study was therefore designed to observe and compare the language and social development of infants 9-18 months of age in various naturally evolving home media and stimulation environments [2,3].

## Materials and methods

Data source and study population

The study was a prospective observational cohort study conducted at the Department of Paediatrics, SRM Medical College Hospital and Research Centre, Tamil Nadu, India. The study observation window was implemented over a six-month period. This was a purely observational design; the investigators did not prescribe interventions or groups, nor did they alter parental behaviour. Instead, the natural behaviours, choices, and habits of the enrolled families were tracked prospectively.

Parents of infants attending routine paediatric outpatient clinic were screened for eligibility.

The inclusion criteria for the study were children aged 9-18 months at baseline, documented daily screen exposure exceeding two hours per day (>2 hours/day), and informed written consent from primary caregivers.

The exclusion criteria were children diagnosed with neurodevelopmental disorders, having sensory impairments (hearing or visual deficits), genetic syndromes, chronic neurological illnesses, or prior formal developmental intervention.

A total of 72 eligible children were enrolled. Over the subsequent six months, families maintained validated monthly lifestyle exposure diaries and participated in brief monthly telephone check-ins to track media exposure and home stimulation habits. 

1. Cohort A (Screen Reduction+High Stimulation; n=36): Caregivers who independently reduced their child’s screen time to less than one hour/day and regularly participated in home language stimulation activities (e.g., interactive reading, singing, storytelling, or naming objects) at least three times a week.

2. Cohort B (Screen Reduction Only; n=36): Caregivers who successfully reduced their child’s screen time to less than one hour/day but did not incorporate regular language stimulation routines into their daily interactions.

Data collection and study variables

The primary outcome was language and social development scores at baseline and at six months follow-up. The assessments were performed by a trained paediatric clinician using the Language Evaluation Scale Trivandrum (LEST) [4] and parent-reported Home Screening Questionnaire (HSQ) [5], a validated developmental screening instrument, optimized for young children.

Potential confounders measured at enrolment included demographic covariates such as parental education levels, household income, and baseline infant media exposure hours.

Ethical considerations

The study protocol was approved by the Institutional Ethics Committee of SRM Medical College Hospital and Research Centre. Ethical clearance number: SRMIEC-ST1225-6001. Written informed consent was obtained from all caregivers.

Data handling and statistical analysis

Data were analysed using SPSS version 26.0 (IBM Corp, Armonk, NY). The continuous variables were presented as mean±standard deviation (SD). Paired and independent t-tests were initially used to assess baseline matching and longitudinal change within groups, respectively.

Due to the observational nature of the cohort design and to control for residual confounding (e.g., socioeconomic status, maternal education), a multivariable linear regression model was applied with the six-month LEST+HSQ subscores as the dependent variables. Significance was considered at a p-value <0.05.

## Results

Baseline characteristics

At baseline, the demographic and developmental profiles of the two cohorts were very similar. The parallel cohorts (Table 1) were well matched at the start of long-term follow-up regarding mean infant age, sex distribution and baseline LEST+HSQ scores and showed no statistically significant differences.

**Table 1:** Baseline demographic and developmental characteristics of the cohorts (n=72) Age and baseline LEST+HSQ scores are presented as mean±standard deviation (SD). An independent t-test was used. Gender is expressed as n(%), analysed using the chi-square test. LEST: Language Evaluation Scale Trivandrum; HSQ: Home Screen Questionnaire. A p-value <0.05 was considered statistically significant.

Variable	Cohort A (Screen Reduction + Stimulation) (n=36)	Cohort B (Screen Reduction Only) (n=36)	t / χ^2 ^value	p-value
Age (months)	13.2±2.5	13.5±2.3	-0.54	0.62
Male, n(%)	19 (52%)	18 (50%)	0.056	0.81
Female, n(%)	17 (48%)	18 (50%)		
Baseline LEST+HSQ Score	42.1±6.3	41.8±6.5	0.20	0.88

Longitudinal multivariable linear regression modelling indicates that, after adjusting for parental education and socioeconomic variations, Cohort A shows significantly higher developmental outcomes than Cohort B, with a 5.40-point increase in language scores and a 3.80-point increase in social scores (p<0.001). The positive effects are robust, with 95% confidence intervals indicating that the advantages for Cohort A are statistically significant and independent of the controlled baseline factors.

Developmental trajectories

Over the six-month prospective observation period, both cohorts displayed positive shifts in overall developmental metrics. However, infants with families that naturally combined screen reduction with high interactive stimulation (Cohort A) achieved a markedly higher change in score compared to those focusing solely on screen reduction (Cohort B) (+16.3 vs. +7.4, p<0.001) (Table 2).

**Table 2:** Intra-cohort mean LEST+HSQ scores from baseline to six months LEST+HSQ scores are presented as mean±SD. LEST: Language Evaluation Scale Trivandrum; HSQ: Home Screen Questionnaire. A paired t-test was used. A p-value <0.05 was considered statistically significant. *Indicates statistical significance.

Cohort	Baseline LEST+HSQ	6-Month LEST+HSQ	Mean Score Change	t value	Within-Group p-value
Cohort A	42.1±6.3	58.4±6.2	+16.3	13.54	<0.001*
Cohort B	41.8±6.5	49.2±6.3	+7.4	3.10	<0.01*

Domain-specific outcomes at follow-up

At the final six-month assessment, domain-specific breakdowns revealed distinct advantages for Cohort A. Cohort A achieved significantly higher scores in both language performance (30.2±3.4 vs. 24.8±3.9, p<0.001) and social behaviour metrics (28.2±3.1 vs. 24.4±3.5, p<0.001) compared to Cohort B (Table 3). These associations remained statistically significant after adjusting for parental education and socioeconomic baseline variations in the multivariable regression model.

**Table 3:** Inter-cohort domain-specific developmental scores at six months Language and social scores are presented as mean±SD. Student unpaired t-test was used. A p-value <0.05 was considered statistically significant. *Indicates statistical significance.

Developmental Outcome Domain	Cohort A (n=36)	Cohort B (n=36)	t value	Unadjusted p-value
Language Score	30.2±3.4	24.8±3.9	6.26	<0.001*
Social Score	28.2±3.1	24.4±3.5	4.87	<0.001*

Multivariable linear regression modelling indicates that, after adjusting for parental education and socioeconomic baseline variations, Cohort A shows significantly higher developmental outcomes than Cohort B, with a 5.40-point increase in language scores and a 3.80-point increase in social scores (p<0.001). The positive effects are robust, with 95% confidence intervals indicating that the advantages for Cohort A are statistically significant and independent of the controlled baseline factors (Table 4).

**Table 4:** Multivariable Regression Analysis A multivariable linear regression model adjusted for parental education and socioeconomic baseline variations. Cohort B serves as the reference group (β=0). ^#^β: Represents the exact mean score increase for a participant being in Cohort A rather than Cohort B when keeping household background constant. 95% Confidence interval (CI): Since neither interval includes 0, the null hypothesis is rejected. *Indicates statistical significance.

Developmental Outcome Domain	Adjusted beta coefficient (β)^#^	Standard error (SE)	95% confidence interval (CI)	Adjusted p-value
Language Score	5.40	0.86	[3.71, 7.09]	<0.001*
Social Score	3.80	0.78	[2.27, 5.33]	<0.001*

Validation of home diaries at the study's conclusion demonstrated that those who naturally prioritized early language enrichment had higher compliance and were able to achieve overall positive language and social developmental scores. High tracking stability and adherence to low screen exposure were maintained by 83% of families in Cohort A, compared to 61% of families in Cohort B.

## Discussion

Early childhood is a unique window of vulnerability and opportunity for neurodevelopment [1, 6]. The human brain is characterized by rapid synaptogenesis, axonal myelination, and cortical architecture mapping in the first three years of life, which are profoundly shaped by environmental stimuli. Such vast neuroplasticity implies that the structural and functional development of brain networks is an experiential manifestation of the child’s immediate surroundings. Language acquisition, joint attention, and socio-emotional milestones are not hardwired genetic sequences; rather, they are active skills cultivated by rich, iterative, bidirectional human communications [6].

The alarming proliferation of handheld digital devices, background televisions, and media-based infant entertainment has radically changed this foundational environmental landscape. In this prospective cohort, when a developing brain is exposed to too much screen media (more than two hours a day), the environment of the developing brain is rapid visual cuts, over-stimulating auditory input, and most importantly, the lack of contingent responsiveness. The findings of this prospective cohort study suggest that children exposed to high screen time at baseline demonstrate evident delay in language and social domains [7].

The most important epidemiological finding in this study is that naturally occurring screen reduction provides a small, baseline trajectory correction, whereas the active, self-selected replacement of screen time with structured caregiver-mediated language stimulation. The removal of the digital device from the environment of the infant simply relieves the infant from negative exposure, but does not provide the positive, linguistically rich and emotionally responsive inputs needed to rapidly repair or accelerate communication pathways [7].

The “displacement hypothesis” is that the primary damage from early screen exposure is not necessarily a direct toxic neurological insult, but the structural displacement of essential real-world experiences. When an infant is passively watching a screen for several hours a day, that infant is losing the same number of hours of human engagement, vocal play, physical exploration, and environmental interaction. Our findings are also in agreement with Nathanson [7], who argued that “children’s attention and learning are greatly improved over passive consumption when parents naturally engage in co-viewing and conversational mediation”. Cohort A was also able to dodge the media distraction trap by moving the family dynamic entirely away from screens and towards high-engagement behaviours.

Madigan et al. [8] found a clear dose-response relationship between screen time in early childhood and poorer performance on developmental screening tests, indicating that more exposure can structurally compromise milestones. Zimmerman et al. [9] reported similar results, stressing that passive media viewing in children under the age of two years is not educational and negatively correlated with expressive vocabulary, while interactive reading and conversation between parent and child are strong positive predictors of linguistic competence.

Furthermore, our findings are in agreement with the work of Tomopoulos et al. [10] who found that infant exposure to media is strongly linked to reducing parent-child verbal interaction and delayed language milestones. Chonchaiya and Pruksananonda [11] reported a significantly higher risk of delayed language acquisition in infants exposed to television before 12 months of age and those watching more than two hours per day.

Children in Cohort B showed marginal improvement when caregivers naturally reduced screen exposure but did not actively implement structured language stimulation routines, probably due to a small natural restoration of basic human contact. Duch et al. [12] reported this phenomenon in their systematic review, concluding that minimizing screen time helps to reduce the deficit but does not fully optimize development if the home stimulation environment remains low.

In contrast, Cohort A increased the quantity and quality of “serve-and-return” exchanges by explicitly naming objects, telling stories interactively and singing rhymes, leading to a statistically significant increase in final language scores (30.2±3.4) versus Cohort B (24.8±3.9).

The difference in social development scores between Cohort A (28.2±3.1) and Cohort B (24.4±3.5) highlights that early language development is intertwined with socio-emotional development. The basis of infant social cognition is joint attention, the shared focus of two individuals on some object or event, achieved by eye-gaze tracking, pointing, and verbal coaching. Joint attention is inherently interactive and forms the cognitive bridge through which a child learns that vocalizations carry social meaning and intention.

Too much screen time disrupts development of joint attention. Passive solo viewing or background media fosters an environment in which the caregiver and child are co-present but psychologically uninvolved. In pedagogical literature, this phenomenon is often referred to as “media distraction,” and it has been shown by Kirkorian et al. [13] to significantly reduce the quantity and emotional quality of parent-child interactions. When a screen is present, caregivers speak fewer words, respond less frequently to cues from the child, and are less emotionally expressive.

The families in Cohort A did shared reading, singing, and direct conversational turn-taking, which are highly concentrated vehicles for joint attention. When a mother points to a picture in a book and names an animal, she is creating a shared mental framework with her infant. This process increases vocabulary, while enhancing emotional bonding, emotional regulation, and social responsiveness.

Neurobiological models, including structural MRI studies by Hutton et al. [14], have shown that increased screen use in preschool-aged children is associated with decreased microstructural integrity of brain white matter tracts that support language and emergent literacy skills. Conversely, enriched reading and interactive environments promote sound myelination of these same tracts. The higher social scores for Cohort A suggest that replacing screens with rich caregiver engagement helps to rewire these social cognition pathways, enabling infants to better read human expressions, interpret social cues, and engage in meaningful communicative play.

The findings of this prospective cohort study have important clinical implications from a public health and preventive paediatric perspective. Historically, clinical advice regarding digital media has been primarily restrictive. Paediatricians usually adhere to the restrictive guidelines of the American Academy of Paediatrics (AAP) [1] and World Health Organization (WHO) [2] and advise parents to enforce strict avoidance of screens for children 18-24 months of age. Although these recommendations are epidemiologically sound, clinical experience suggests that negative commands (“do not allow screen time”) often result in parental guilt but not actionable, real-world alternatives, and also poor compliance.

The findings of this study suggest that counselling needs to move from a model of pure restriction to a model of active behavioural substitution. Families who self-selected to seek out language stimulation techniques were significantly more stable and compliant with screen reduction (83% vs. 61%), indicating that giving parents a clear positive script is behaviourally more effective than simply removing the device. If parents have practical, readily available alternatives such as singing during a bath, naming ingredients during meal prep, or reading a picture book before bed, the digital device drops out as the default soothing or passive babysitter.

Furthermore, this model is highly scalable and cost-effective, making it highly relevant for low- and middle-income countries (LMICs) and resource-limited tertiary care settings. It requires no specialized technology, software, or expensive clinical equipment. Teaching parents basic language stimulation during routine immunization or wellness visits is a low-cost public health strategy that can be easily incorporated into primary paediatric care to ameliorate the modern epidemic of digital-media-related developmental delays.

Strength of the study

This is a study of naturally occurring parental decisions in the home environment in order to measure real-world behavioural dynamics. The objective reliability of the data is achieved through the use of LEST, which is a standardized, clinically validated tool optimised for early developmental screening.

Limitations

A cohort study is subject to selection bias as opposed to randomized controlled trials where randomization balances unmeasured confounders. Parents who are naturally inclined to spend more time reading, singing, and engaging with their infants (Cohort A) may have different underlying demographic characteristics compared to those who are not (Cohort B). Increased parental education, increased household income, reduced maternal stress, and a more resourceful home environment are all associated with less child screen exposure and increased verbal engagement. To minimize limitation, our analysis used multivariable linear regression modelling for the mathematical control of baseline socioeconomic status and maternal education levels.

Additionally, the data on the duration of screen time and frequency of language stimulation were based on parental self-report in lifestyle diaries and telephone interviews, which may be subject to recall and social desirability biases.

Finally, while the six-month follow-up is sufficient for the detection of early developmental acceleration, it is too short to evaluate the long-term sustainability of these gains or to track their impact on later school readiness, executive function and academic literacy.

## Conclusions

For infants and children between nine and 18 months with a background of high media exposure, the natural, self-selected addition of home language stimulation plus screen reduction is associated with significantly better language and social development outcomes than screen restriction alone.

Routine developmental counselling during paediatric outpatient and immunization visits can be an effective opportunity to promote language-rich home environments alongside screen-time guidance. Simple, home-based language stimulation strategies are feasible, inexpensive, and potentially scalable public health interventions, particularly in resource-limited settings.

## References

[1] (2016). Media and young minds. Pediatrics.

[2] (2019). World Health Organization. Guidelines on physical activity, sedentary behaviour and sleep for children under 5 years of age. Geneva: WHO. https://www.who.int/publications/i/item/9789241550536.

[3] Christakis DA (2009). The effects of infant media usage: what do we know and what should we learn?. Acta Paediatr.

[4] Nair MK, Nair GH, Mini AO, Indulekha S, Letha S, Russell PS (2013). Development and validation of language evaluation scale Trivandrum for children aged 0-3 years - LEST (0-3). Indian Pediatr.

[5] Nair MK, Prasanna GL, Jeyaseelan L, George B, Resmi VR, Sunitha RM (2009). Validation of Home Screening Questionnaire (HSQ) against Home Observation for the Measurement of Environment (HOME). Indian Pediatr.

[6] Vanderloo LM (2014). Screen-viewing among preschoolers in childcare: a systematic review. BMC Pediatr.

[7] Nathanson AI (2001). Parent and child perspectives on the presence and meaning of parental television mediation. Journal of Broadcasting and Electronic Media.

[8] Madigan S, Browne D, Racine N, Mori C, Tough S (2019). Association between screen time and children's performance on a developmental screening test. JAMA Pediatr.

[9] Zimmerman FJ, Christakis DA, Meltzoff AN (2007). Associations between media viewing and language development in children under age 2 years. J Pediatr.

[10] Tomopoulos S, Dreyer BP, Berkule S, Fierman AH, Brockmeyer C, Mendelsohn AL (2010). Infant media exposure and toddler development. Arch Pediatr Adolesc Med.

[11] Chonchaiya W, Pruksananonda C (2008). Television viewing associates with delayed language development. Acta Paediatr.

[12] Duch H, Fisher EM, Ensari I, Harrington A (2013). Screen time use in children under 3 years old: a systematic review of correlates. Int J Behav Nutr Phys Act.

[13] Kirkorian HL, Pempek TA, Murphy LA, Schmidt ME, Anderson DR (2009). The impact of background television on parent-child interaction. Child Dev.

[14] Hutton JS, Dudley J, Horowitz-Kraus T, DeWitt T, Holland SK (2020). Associations between screen-based media use and brain white matter integrity in preschool-aged children. JAMA Pediatr.

